# Comparison of several sequence-based association methods in pedigrees

**DOI:** 10.1186/1753-6561-8-S1-S48

**Published:** 2014-06-17

**Authors:** George Mathew, Varghese George, Hongyan Xu

**Affiliations:** 1 Department of Mathematics, Missouri State University, 901 South National Avenue, Springfield, Missouri 65897, USA; 2 Department of Biostatistics & Epidemiology, Georgia Regents University, 1469 Laney Walker Boulevard, Augusta, Georgia 30912-4900, USA

## Abstract

Genome-wide association studies are very powerful in determining the genetic variants affecting complex diseases. Most of the available methods are very useful in detecting association between common variants and complex diseases. Recently, methods to detect rare variants in association with complex diseases have been developed with the increasingly available sequencing data from next-generation sequencing. In this paper, we evaluate and compare several of these recent methods for performing statistical association using whole genome sequencing data in pedigrees. Specifically, functional principal component analysis (FPCA), extended combined multivariate and collapsing (CMC) method for families, a generalized T^2 ^method, and chi-square minimum approach were compared by analyzing all the genetic variants, common and rare, of both the real data set and the simulated data set provided as part of Genetic Analysis Workshop 18.

## Background

With advances in genotyping technologies, genome-wide association studies (GWAS) became a very popular procedure to identify disease genes and other traits by conducting statistical tests on many thousands of single-nucleotide polymorphisms (SNPs). The procedure has great potential for discovering genetic variants influencing complex diseases. However, these procedures have discovered loci that account only for a small percentage of phenotypic variance [1]. One of the reasons for this difficulty may be that rare variants might explain disease susceptibility [2-4]. Recently, several methods have been developed to determine the influence of rare variants on complex diseases. These methods differ from the traditional methods of testing where the focus has been on individual common variants. It is understood that those variants with a population frequency greater than 5% are considered to be common variants, those with less than 1% population frequency as rare variants, and the rest as low-frequency variants [4]. The common variants are believed to be from distant ancestors, whereas rare variants are from recent ancestors [5]. Most of these methods assume the individuals are independent and are designed for population-based data. Only recently have several methods been developed that can perform statistical association of sequence data in pedigrees. In this paper, we used functional principal component analysis (FPCA) [4], the generalized T^2 ^approach [4], the combined multivariate and collapsing (CMC) test for family data [2,4], and the chi-square minimum approach for family data [4] to analyze association of the dichotomous hypertension trait with all genetic variants, common and rare, of the real data set and all replicates of chromosome 3 of the simulated data set provided by Genetic Analysis Workshop 18 (GAW 18) [6]. We compared the results to assess the merits of these methods.

## Methods

An extension of the generalized *T*^2 ^test [7] for family-based association studies is provided by Zhu and Xiong [4]. The test statistic is given by $T_{F}^{2}=\frac{T^{2}}{P_{corr}}$, where *T*^2 ^is the generalized *T*^2 ^statistic [7], and *P_corr _*[4, p. 1030] is the correction factor to account for the familial correlation in the pedigree data. A similar extension of the CMC test is also developed and is provided by equation (15) in Zhu and Xiong [4]. The test statistic is given by $T_{CMCF}=\frac{T_{CMC}}{P_{corr}}$, where *T_CMC _*is the CMC statistic for the population-based association test, and *P_corr _*is the correction factor to adjust *T_CMC _*statistic so that it is valid for pedigree data. The FPCA statistic for the population-based association test in Luo et al [8] also has a similar extension for family data [4], and is given by $T_{FPCAF}=\frac{T_{FPCA}}{p_{corr}}$, where *T_FPCA _*and *P_corr _*are defined as in the previous test statistics for pedigree data. Also, $T_{F}^{2}$, *T_CMCF_*, and *T_FPCAF _*have chi-square distributions [4]. The chi-square minimum statistic chooses the minimum of the *p*-values from the individual chi-square tests for each genetic variant from a genomic region. The chi-square minimum statistic (Chi_min) adjusts for relatedness of pedigree members using *P_corr _*[4].

We applied the above 4 methods to analyze the real data set from all odd-numbered chromosomes using hypertension status at exam 1 as the phenotype. The genotypes at each variant are coded as 0, 1, or 2 for aa, Aa, and AA, where allele A is the minor allele. The start and end boundary of all the human genes were obtained from hg19 genome assembly at NCBI. The genetic variants within 1 gene were analyzed together as each gene is 1 natural functional unit. For the FPCA method, if there are too few genetic variants, that is, less than 3, the estimate of the functional relation of the allele counts across the genetic variants will be far off. Consequently, genes with fewer than 3 genetic variants were not analyzed. The significant genes from the 4 methods were then compared with the findings from previous GWAS for genes associated with blood pressure.

To examine the type I error rate and power of the 4methods, we applied these methods to the 200 replicates of the simulated data set and analyzed the data from chromosome 3. As in the real data analysis, we chose the hypertension status at exam 1 as the phenotype.

## Results

**Figure 1:**
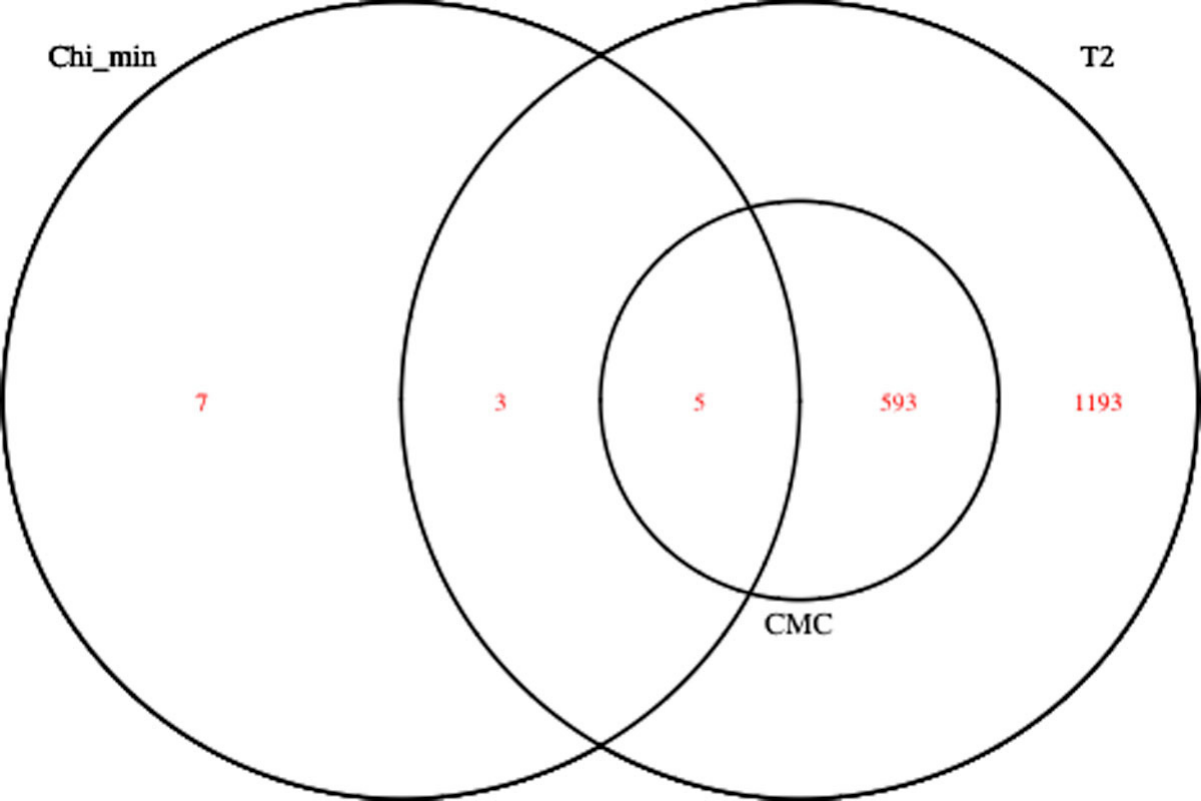
Venn diagram showing overlaps of the significant genes from Chi_min, CMC, and T^2 ^at 4.7 × 10^−6 ^level from the analysis all odd-numbered chromosomes of the real data set.

Table 1 gives the number of significant genes at several α levels from the real data analysis for a total of 10,580 genes from the odd-numbered human chromosomes. FPCA method finds the fewest number of significant genes compared to the other 3 methods. Chi_min finds the highest number of significant genes at 0.05 and 0.01 levels. However, T^2 ^finds more significant genes at lower significance levels (0.001, 0.0001, and 4.7 × 10^−6^). The number of genessignificant at the 4.7 × 10^−6 ^level by the FPCA, Chi_min, CMC, and T^2 ^methods are 0, 15, 598, and 1794, respectively. Figure 1 is a Venn diagram showing overlaps of the significant genes from Chi_min, CMC, and T^2 ^at 4.7 × 10^−6 ^level. It is interesting to note that all 598 significant genes found by CMC overlap with those found by T^2^.

**Table 1:** Number of significant genes out of 10,580 genes in the odd-numbered human chromosomes of the real data set at various significance levels

Method	Significance level				
	
	0.05	0.01	0.001	0.0001	4.7 × 10^−6^
FPCA	158	33	3	1	0
Chi_min	8321	5123	1402	172	15
T^2^	3902	3079	2436	2050	1794
CMC	2083	1329	907	717	598

The number of significant genes presented in Table 1 will contain false positives, as with any statistical test. To get an idea of the number of "true findings," we compared our results with those findings of GWAS for blood-pressure-associated genes. We performed a comprehensive literature review, and 84 genes were identified as being associated with blood pressure from GWAS. Table 2 shows the number of overlapped genes between our analysis and the GWAS findings.

**Table 2:** Number of overlapped genes associated with blood pressure from GWAS findings at various significance levels

Method	0.05	0.01	0.001	0.0001	4.7 × 10^−6^
FPCA	0	0	0	0	0
Chi_min	36	23	9	1	0
T^2^	20	18	14	12	12
CMC	12	10	7	5	4

We analyzed chromosome 3 of the simulated data set. There are a total of 1120 genes on chromosome 3, of which 30 were used for causal variants of hypertension in the simulation model. The remaining 1090 were assumed to be unrelated to the disease and are used only for calculating type I error rate. The linkage disequilibrium (LD) between the genetic variants from these groups of 1090 genes and 30 genes were analyzed with Haploview [9] and no significant LD was found. Table 3 lists the type I error rates from the analysis of all 200 replicates by all 4 methods at various significance levels.

**Table 3:** Type I error probability estimates by FPCA, Chi_min, T^2^, and CMC methods from all 200 replicates of chromosome 3 of the simulated data set

*α*	FPCA	Chi_min	T^2^	CMC
0.05	0.02567	0.86265	0.05061	0.04763
0.01	0.00656	0.61023	0.01202	0.00908
0.001	0.00096	0.25719	0.00093	0.00136
0.0001	0.00016	0.07272	0.00013	0.00011

The analysis of the 30 positive genes is used to calculate the power of the various methods. Table 4 lists the estimates of the power by the various methods.

**Table 4:** Estimates of power by FPCA, Chi_min, T^2^, and CMC methods from all 200 replicates of chromosome 3 of thesimulated data set

*α*	FPCA	Chi_min	T^2^	CMC
0.05	0.045	0.95433	0.6585	0.338
0.01	0.01883	0.72117	0.57717	0.24583
0.001	0.00483	0.33233	0.50117	0.18083
0.0001	0.00117	0.09667	0.448	0.14233

## Discussion

With the increasingly available sequence data from the next-generation sequencing technologies, it is important for a statistical association method to handle both common and rare genetic variants. It is also important for these methods to handle data from pedigrees because rare genetic variants are enriched in families with multiple affected individuals, which could confer more statistical power. From our analysis of the real data, T^2 ^seems to be a better method than the other 3 methods because it finds more significant genes at low significance levels. At the Bonferroni corrected *p*-value of 4.7 × 10^−6 ^, T^2 ^identified the genes *CASZ1, ADAMTS8, NUCB2, ABCC8, SLC4A7, MAP4, CASR, EBF1, PLEKHA7, SOX6, ULK4*, and *MECOM*. The last 4 genes were also identified by the CMC method. All the genes mentioned above were found to be associated with blood pressure, in particular *ULK4 *and *PLEKAH7 *by Levy et al [10], and *MAP4 *by Wain et al [11].

As with GWAS, we need to keep a low significance level to account for multiple testing. We note from the analysis of the simulated data that FPCA has empirical type I error rate much less than the nominal value, making it very conservative. The Chi_min method has inflated type I error rate. The type I error rates by T^2 ^and CMC are close to the nominal value. Also, T^2 ^has better power than CMC, which is consistent with the result from the real data.

From the analysis of the data sets we find that T^2 ^is a better method, which is different from the findings of Zhu and Xiong [4], which suggest that FPCA is a better procedure. There are 2 possible reasons why FPCA performs less well here. One reason may be that the SNPs in the genes are sparse. If there are too few SNPs in 1 gene, the FPCA may not perform well because the number of SNPs is not enough to estimate the function describing the allele counts across the SNPs in the gene. A second reason may be that the assumption of a smooth function of the allele counts across the SNPs for the FPCA may not hold for the GAW 18data sets. We observed a large overlap between the results of CMC and T^2^. This mainly comes from the fact that CMC uses the T^2 ^approach with common variants. There is also a tendency to pick up more genes with more variants for both CMC and T^2 ^methods.

## Conclusions

From the analysis results of both real and simulated data, T^2 ^is a preferable method for pedigree-based association studies with whole-genome sequencing data because it controls the false positive rate and is more powerful than the other two methods with similar type I error rates.

## Competing interests

The authors declare that they have no competing interests.

## Authors' contributions

HX conceived of the study, performed the analysis, and helped to draft the manuscript. GM performed the analysis and helped to draft the manuscript. VG participated in the design and coordination of the study. All authors read and approved the manuscript.
